# IMPLEMENTING EPIC-VR IN HEALTH CARE: ALIGNING VIRTUAL REALITY TRAINING WITH ORGANIZATIONAL CONTEXT AND READINESS

**DOI:** 10.1093/geroni/igae098.0072

**Published:** 2024-12-31

**Authors:** Marie Savundranayagam, Grace Norris, Grace Malheiro, Annette Schumann, Jennifer Campos, Joseph Orange

**Affiliations:** Western University, London, Ontario, Canada; Western University, London, Ontario, Canada; Western University, London, Ontario, Canada; Western University, London, Ontario, Canada; The KITE Research Institute, University Health Network, Toronto, Ontario, Canada; Western University, London, Ontario, Canada

## Abstract

Be EPIC-VR is a virtual reality (VR)-based person-centered communication training for frontline healthcare workers in dementia care. The current study investigated factors influencing the successful implementation of Be EPIC-VR in home care and long-term care, using the Consolidated Framework for Implementation Research. Participants included eight managers from four care settings. Managers were chosen for their decision-making roles in enabling their teams to take Be EPIC-VR. Semi-structured interviews were conducted before and after Be EPIC-VR’s implementation and analyzed using framework analysis. Two themes emerged: organizational context and organizational readiness. Organizational context had two subthemes: increased training needs and staffing resources. Managers identified dementia-specific training needs evolving from limited training during the COVID-19 pandemic. Staffing-related resources, such as scheduling and backfilling, were essential for implementation. This organizational context shaped the organizational readiness for Be EPIC-VR, which was evidenced by three subthemes: relative priority for Be EPIC-VR, relative advantage of Be EPIC-VR, and tailoring strategies for successful implementation. Managers prioritized Be EPIC-VR’s implementation because it aligned with organizational goals to support communication and address responsive behaviours. Managers reported on the relative advantage of Be EPIC-VR compared with existing training programs, emphasizing its interactive VR simulations, the ability to practice new skills in a safe environment, and personalized feedback. Tailored strategies affecting implementation included having sufficient resources to support remote delivery, piloting with small groups, and promoting an openness to VR technology. The findings underscore the value of aligning innovations with organizational goals and tailoring implementation plans to specific organizational contexts.

